# Adsorption and Desorption of Nickel(II) Ions from Aqueous Solution by a Lignocellulose/Montmorillonite Nanocomposite

**DOI:** 10.1371/journal.pone.0117077

**Published:** 2015-02-03

**Authors:** Xiaotao Zhang, Ximing Wang

**Affiliations:** 1 College of Science, Inner Mongolia Agricultural University, Hohhot, PR China; 2 College of Material Science and Art Design, Inner Mongolia Agricultural University, Hohhot, PR China; University of Calcutta, INDIA

## Abstract

A new and inexpensive lignocellulose/montmorillonite (LNC/MMT) nanocomposite was prepared by a chemical intercalation of LNC into MMT and was subsequently investigated as an adsorbent in batch systems for the adsorption-desorption of Ni(II) ions in an aqueous solution. The optimum conditions for the Ni(II) ion adsorption capacity of the LNC/MMT nanocomposite were studied in detail by varying parameters such as the initial Ni(II) concentration, the solution pH value, the adsorption temperature and time. The results indicated that the maximum adsorption capacity of Ni(II) reached 94.86 mg/g at an initial Ni(II) concentration of 0.0032 mol/L, a solution pH of 6.8, an adsorption temperature of 70°C, and adsorption time of 40 min. The represented adsorption kinetics model exhibited good agreement between the experimental data and the pseudo-second-order kinetic model. The Langmuir isotherm equation best fit the experimental data. The structure of the LNC/MMT nanocomposite was characterized by X-ray diffraction (XRD) and transmission electron microscopy (TEM), whereas the adsorption mechanism was discussed in combination with the results obtained from scanning electron microscopy (SEM), energy-dispersive X-ray spectroscopy (EDX), and Fourier-transform infrared spectroscopy analyses (FTIR). The desorption capacity of the LNC/MMT nanocomposite depended on parameters such as HNO_3_ concentration, desorption temperature, and desorption time. The satisfactory desorption capacity of 81.34 mg/g was obtained at a HNO_3_ concentration, desorption temperature, and desorption time of 0.2 mol/L, 60 ºC, and 30 min, respectively. The regeneration studies showed that the adsorption capacity of the LNC/MMT nanocomposite was consistent for five cycles without any appreciable loss in the batch process and confirmed that the LNC/MMT nanocomposite was reusable. The overall study revealed that the LNC/MMT nanocomposite functioned as an effective adsorbent in the detoxification of Ni(II)-contaminated wastewater.

## Introduction

Water contamination caused by heavy-metal ions generated from alloys, pigments, electroplating, mining, metallurgical activities, nuclear power plant operations, aerospace industries, electrical contacts, printing, and the manufacture of paper, rubber, plastics, and batteries [1, 2] is a global problem receiving worldwide attention. The extended persistence of water contamination in biological systems and the tendency to bioaccumulate while moving up the food chain is a serious threat to human health, living resources, and ecological systems [3, 4]. Nickel is a non-biodegradable toxic metal ion present in wastewater, and the presence of Ni(II) ions in drinking water in concentrations that exceed the permissible limit of 0.02 mg/L may cause adverse health effects such as anemia, diarrhea, encephalopathy, hepatitis, lung and kidney damage, gastrointestinal distress, pulmonary fibrosis, renal edema, skin dermatitis, and central nervous system dysfunction [5–9]. Therefore, the treatment of industrial effluents rich in Ni(II) before discharge is necessary. Increased awareness of heavy metal toxicity has led to a dramatic increase in research on various technologies to clean targeted water environments. Different methods, such as ion exchange, reduction, flocculation, reverse osmosis, membrane filtration, and precipitation, have been investigated for the removal of Ni(II) ions from aqueous solutions; however, most of these methods are expensive or generate harmful waste products [10–14]. Adsorption is an effective technique with many advantages, including highly efficient extraction of metals, even from dilute solutions; minimization of secondary wastes; and cost-effectiveness. Because of economic considerations, natural polymeric materials [15] are a promising alternative as adsorbents for wastewater treatment.

Lignocellulose (LNC) is an ideal natural adsorbent for the removal of heavy-metal ions because of its specific structural characteristics [16]. LNC, which is a renewable polymer, is widely distributed in plants and is primarily composed of cellulose, hemicellulose, and lignin [17]. Moreover, a variety of reactive functional groups (FGs), including phenolic, hydroxyl, carboxyl, and other FGs, exist in its three-dimensional structure and act as active sites for the adsorption of heavy metal ions [18]. However, because of its polydispersity and amorphous structure, LNC is not suitable for industrial applications. Recently, clays have been considered one of the most appropriate and inexpensive adsorbents for the removal of heavy metal ions from wastewater. Among the clays studied, montmorillonite (MMT), a type of silicate mineral with a nanolamellar structure, is a low-cost candidate; however, because of MMT’s low affinity, swelling and dispersion in water [19], it adsorbs heavy-metal ions only on the external broken bonds on its surface, which are present in very small numbers [20]. Therefore, to improve the adsorption capacity of heavy metal ions on the MMT surface, the chemical modification of MMT was explored in this study.

The objective of the present study was to evaluate the efficiency of lignocellulose/montmorillonite (LNC/MMT) nanocomposites in the removal of Ni(II) ions from model dilute aqueous solutions intended to mimic low-metal-concentration wastewaters. The effects of environmental parameters such as the initial Ni(II) concentration, pH level, adsorption temperature, and adsorption time on Ni(II) uptake were investigated, and the desorption of Ni(II) adsorbed onto the LNC/MMT nanocomposite was also examined. Different kinetic models were analyzed using pseudo-first-order and pseudo-second-order models. The Langmuir and Freundlich models are commonly used to describe adsorption equilibrium data and were applied to the experimental results. The behavior of the LNC/MMT nanocomposite was studied in continuous mode during adsorption-desorption recirculation experiments performed at the laboratory scale to investigate the feasibility of a cost-effective process for industrial applications. Moreover, the structure and presence of functional groups on the LNC/MMT nanocomposite, which may play an important role in the adsorption process, were confirmed by XRD, TEM, SEM-EDX, and FTIR to elucidate the interaction mechanism between Ni(II) ions and the LNC/MMT nanocomposite.

## Materials and Methods

### Materials

LNC (SAM-100) was purchased from Beijing Huaduo Biotech, Ltd., China. MMT (CEC = 100 meq·(100 g)^−1^) was purchased from Zhejiang Feng Hong Clay Chemical Co., China. MMT was washed using deionized water, dried overnight at 70°C, milled using a blender and finally sieved to 200 mesh particle size. Ni(NO_3_)_2_·6H_2_O was purchased from Shanghai Jinshan Chemical Co., China. All other chemicals and reagents used in the study were of analytical grade and used without further purification. All solutions were prepared using deionized water.

### Methods

**Reagents**. A stock Ni(II) solution (1 mol/L) was prepared by dissolving a weighed quantity of analytical-grade Ni(NO_3_)_2_·6H_2_O in distilled deionized water and diluting to a concentration of 1 mol/L. In this study, the 1 mol/L Ni(II) stock solution was subsequently used to prepare solutions with initial Ni(II) concentrations of 0.0025 to 0.0036 mol/L, and the pH value of each Ni(II) solution was adjusted to the desired value using 0.1 mol/L HCl or NaOH solution. Fresh diluted solutions were used for each experiment.

**Adsorbents**. The LNC/MMT nanocomposite was prepared as follows: a target amount of LNC was dissolved in NaOH solution [1:30 ratio of LNC weight (g) to NaOH volume (mL)] in batches and magnetically stirred, forming a uniform LNC suspension. A suspension of MMT (1.0 g suspended in 30 mL of distilled deionized water) was subsequently stirred for 0.5 h at 500 rpm, followed by addition to the LNC-NaOH suspension. The reaction mixture was heated to 60°C and stirred for 6 h, centrifuged using a centrifuge (H-2050R, Changsha Xiangyi Centrifuge Instrument Co., Ltd, Changsha, China) and washed with HCl and NaOH solutions until the pH of the supernatant was 7.0. The pH of the solution was measured using a pH meter (PB-10, Sartorius Scientific Instruments Co., Ltd, Beijing, China). Finally, the obtained material was dried under vacuum at 105°C in a vacuum oven (DZF-6210, Shanghai Yiheng Scientific Instrument Co., Ltd, Shanghai, China) for 5 h until a constant weight was achieved. All samples were ground and sieved to a 200 mesh particle size and were subsequently stored in an airtight plastic container until used for the specific experiments.

**Characterization**. X-ray diffraction (XRD) analysis of the powdered samples was performed using a PANanalytical X’pert PRO X-ray powder diffractometer equipped with a Cu anode operated at 40 kV and 30 mA; the samples were scanned at a rate of 3°/min from 4° to 18°. TEM image analyses of the samples were performed on a JEM-2010 electron microscope operated at 200 kV. The surface morphology of the samples was recorded by scanning electron microscopy (SEM, HITACHI S-4800, Japan). All of the samples were fixed onto aluminum stubs and coated with gold prior to SEM observation; the surface elemental analyses of Ni(II) on the LNC/MMT nanocomposite were conducted by energy-dispersive X-ray spectroscopy (EDX, HITACHI S-4800, Japan). The infrared spectra of the unloaded and Ni(II)-loaded LNC/MMT composites were obtained using Fourier-transform infrared spectrophotometry (FTIR, Thermo Nicolet NEXUS Thermo Fisher Scientific, Waltham, MA, USA). The LNC/MMT nanocomposite was crushed, ground, and encapsulated in KBr pellets to prepare translucent sample disks; FTIR spectra were recorded in the range of 4000–500 cm^-1^ by averaging 20 scans at a maximum resolution of 4 cm^-1^.

**Adsorption experiments**. LNC/MMT (0.1000 g) was accurately weighed and added to 50 mL of the Ni(II) solution. The suspension was stirred at a uniform speed of 120 rpm in a thermostatic shaker (SHA-C). Throughout the course of the experiments, the pH of each Ni(II) solution was adjusted to a constant value by periodic shaking after the addition of 0.1 mol/L HCl or NaOH solution. When adsorption equilibrium was reached, the mixtures were centrifuged at 6000 rpm for 5 min. Then, the Ni(II) concentration of the upper fluid was determined by the dimethylglyoxime method [21]. The absorbance of the wine-red-to-brown-colored Ni(II) complex using dimethylglyoxime was measured at 470 nm in a double beam UV-visible spectrophotometer (TU-1901, Beijing Purkinje General Instrument Co., Ltd. China). The adsorption experiments were conducted by varying the initial Ni(II) concentrations, pH values, adsorption temperatures, and adsorption times. Taking into account the experimental errors and based on the average values, three independent replicates confirmed that the results of the Ni(II) removal experiments were reproducible under the same conditions. The amount of Ni(II) adsorbed at time *t* by the LNC/MMT nanocomposite, which represents the Ni(II) adsorption capacity, was calculated according to the following mass-balance relationship [22]:

$$q_{t,1}=\frac{(C_{0}-C_{t,1})V_{1}\times 58.7}{m_{1}}$$ (1)

where *q_t,1_* (mg/g) is the capacity of adsorption at time *t* (min); *C_0_* and *C_t,1_* (mol/L) are the Ni(II) initial and final concentrations at time *t* (min), respectively; *V_1_* (mL) refers to the volume of Ni(II); and *m_1_* (g) is the mass of adsorbent. To calculate *q_t,1_*, it was assumed that no Ni(II) ions were lost via any other mechanism such as volatilization, sorption to the glassware, or degradation.

**Desorption experiments.** HNO_3_ (0.1 mol/L) was used as the elution reagent, and the experiments were conducted as follows. The Ni(II)-loaded LNC/MMT nanocomposite (0.1000 g) was accurately weighed and subsequently dissolved in 50 mL of HNO_3_ solution at different concentrations. The mixture was placed in an ultrasonic cleaning machine (KS-300EI). During the desorption experiments, the Ni(II) ions were replaced by hydrogen ions from the aqueous HNO_3_. After the desorption equilibrium reached the target temperature, the suspension was centrifuged and the concentrations of the desorbed Ni(II) solutions were determined. Taking into account the experimental errors, experiments were performed in triplicate; the reproducibility of the results was within ±3%. The desorption capacity of the Ni(II)-loaded LNC/MMT nanocomposite was calculated according to the following equation [23, 24]:

$$q_{t,2}=\frac{C_{t,2}V_{2}\times 58.7}{m_{2}}$$ (2)

where *q_t,2_* (mg/g) is the amount of Ni(II) desorbed at time *t* (min), *C_t,2_* (mol/L) is the concentration of Ni(II) in the desorbed solution at time *t* (min), *V_2_* (mL) is the total volume of the solution, and *m_2_* (g) is the mass of the adsorbent after the adsorption of Ni(II).

**Recycling and Regeneration Experiments**. To investigate the reusability of the LNC/MMT nanocomposite, repeated batch adsorption-desorption experiments were performed. After the first batch reaction, the LNC/MMT nanocomposite was washed with distilled deionized water to remove the remaining acid and was dried in a vacuum oven at 70°C in preparation for the next adsorption of Ni(II). The adsorption-desorption capacities of the LNC/MMT nanocomposite for Ni(II) were determined using a TU-1901. The regenerated LNC/MMT nanocomposite was used in six consecutive cycles of adsorption-desorption experiments, and the experimental results indicate that losses in the activity of the LNC/MMT nanocomposite were negligible for the first 5 cycles.

## Results and Discussion

### Adsorption Studies

**Fig 1:**
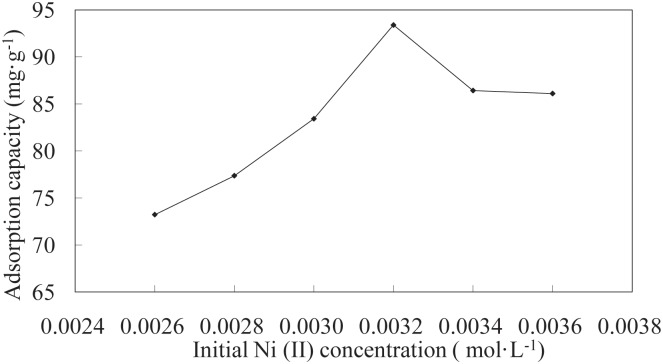
Effect of the initial Ni(II) concentration on adsorption capacity of LNC/MMT. Adsorption experiments-sample dose, 0.1000 g; pH, 6.8; temperature, 70°C; time, 40 min.

**Effect of initial Ni(II) concentration on adsorption**. The initial Ni(II) concentration serves as an important driving force for overcoming mass transfer resistance of Ni(II) between the aqueous and solid phases. The effects of different initial Ni(II) concentrations on the LNC/MMT nanocomposite adsorption capacity are shown in Fig. 1. The adsorption capacity of the LNC/MMT nanocomposite toward Ni(II) first increased and then remained constant with increasing initial Ni(II) concentration. This result was observed because higher Ni(II) concentrations result in an increased concentration gradient, which, leads to a higher probability of collision among Ni(II) ions and the active adsorption sites on the LNC/MMT nanocomposite, thereby increasing adsorption capacity. With further increases in Ni(II) concentration, the adsorption capacity remained constant because the active adsorption sites became saturated [25]. Therefore, Ni(II) with an initial concentration of 0.0032 mol/L was chosen as the ideal initial concentration for all subsequent experiments.

**Fig 2:**
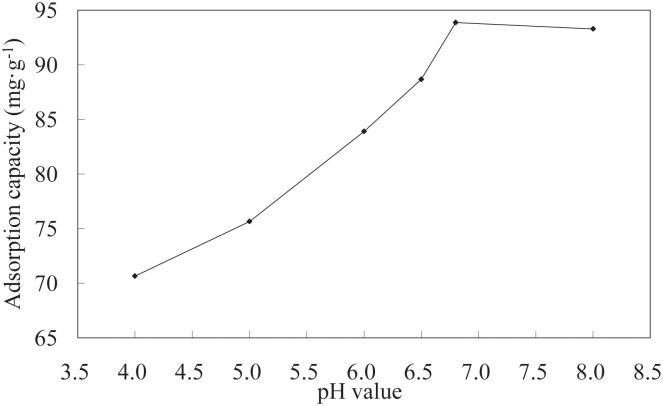
Effect of pH on adsorption capacity of LNC/MMT. dsorption experiments-sample dose, 0.1000 g; the initial Ni(II) concentration, 0.0032 mol/L; temperature, 70°C; time, 40 min.

**Effect of pH on adsorption**. The pH of the Ni(II) solution is an important factor for determining the adsorption of Ni(II) on the surface of the LNC/MMT nanocomposite. The effects of pH on the adsorption capacity are shown in Fig. 2. These results indicate that the adsorption capacity of Ni(II) first increased and then became constant with increasing pH. A pronounced dependence of Ni(II) adsorption on the solution pH was observed. This adsorption behavior may result from the strong electrostatic repulsion between the positively charged LNC/MMT surface and the Ni(II) ions in solution, which hinders the metal-binding process. With increasing pH, the repulsive interactions decreased and the extent of Ni(II) adsorption increased, presumably because of an ion-exchange mechanism between the surface protons and the Ni(II) ions [26]. The optimum pH value for adsorption was determined to be 6.8, and this pH was used in subsequent studies.

**Fig 3:**
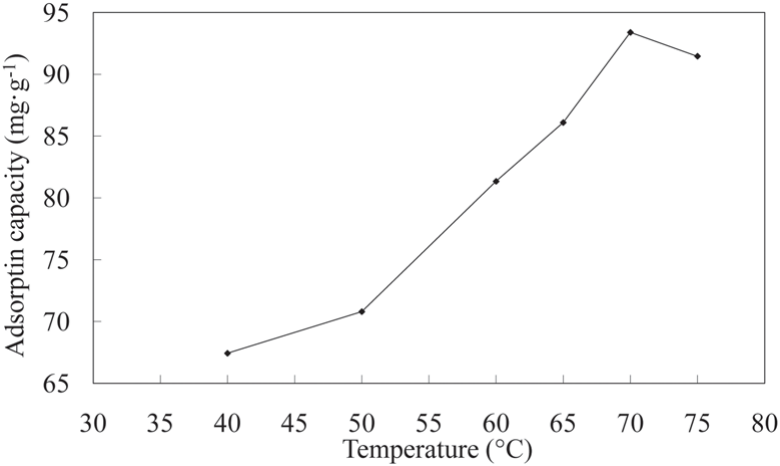
Effect of temperature on adsorption capacity of LNC/MMT. Adsorption experiments-sample dose, 0.1000 g; the initial Ni(II) concentration, 0.0032 mol/L; pH, 6.8; time, 40 min.

**Effect of temperature on adsorption**. The plot of LNC/MMT Ni(II) adsorption capacity vs. temperature is shown in Fig. 3. The Ni(II) adsorption capacity increased as the temperature was increased from 40 to 75°C because of the increased diffusion rate of Ni(II) ions across the external boundary layer and within the pores of the LNC/MMT nanocomposite and because of a decrease in the thickness of the boundary layer surrounding the adsorbent. Consequently, the mass transfer resistance of Ni(II) ions in the boundary layer decreased. Furthermore, at higher temperatures, the energy of the system facilitated the binding of Ni(II) to the surface of the LNC/MMT nanocomposite, indicating that the adsorption of Ni(II) ions on the LNC/MMT nanocomposite surface was controlled by an endothermic process [27]. Therefore, an adsorption temperature of 70°C was chosen as the ideal temperature.

**Fig 4:**
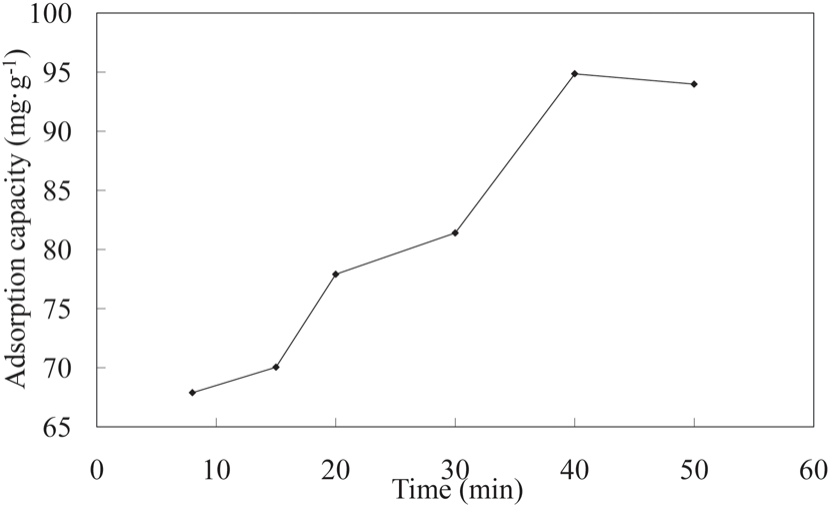
Effect of time on adsorption capacity of LNC/MMT. Adsorption experiments-sample dose, 0.1000 g; the initial Ni(II) concentration, 0.0032 mol/L; pH, 6.8; temperature, 70°C.

**Effect of time on adsorption**. The effects of different adsorption times on the LNC/MMT nanocomposite adsorption capacity toward Ni(II) are shown in Fig. 4. At prolonged adsorption times, the Ni(II) adsorption capacity of the LNC/MMT nanocomposite initially increased rapidly and then decreased slowly because the surface of the LNC/MMT composite was covered with a large quantity unsaturated functional groups. Ni(II) ions were adsorbed by diffusing into the microporous adsorbent; a complex was formed within the active sites of the adsorbent, thus resulting in a sharp adsorption equilibrium that decreased with the saturation of the functional groups on the LNC/MMT surface [28]. Therefore, the optimum adsorption time of 40 min was selected for all further experiments.

### Kinetic Studies

**Fig 5:**
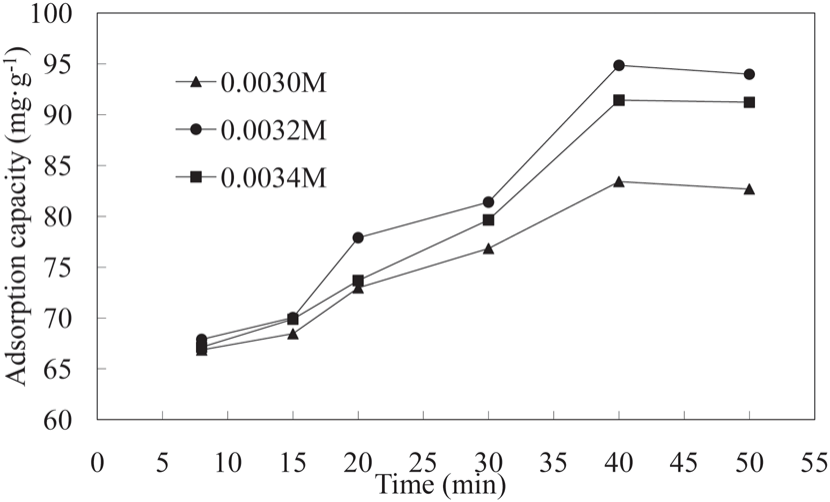
Effects of time on adsorption by LNC/MMT at various initial Ni(II) concentrations.

The dependence of the reaction rate on the initial Ni(II) concentration was investigated by assuming pseudo-first-order and pseudo-second-order models. Fig. 5 showed the effect of time on the LNC/MMT nanocomposite adsorption capacities at various initial Ni(II) concentrations. As evident in the figure, *q_t_* increased significantly with increasing time *t* (min) during the initial stage and then slowly decreased, followed by a continuous increase at time *t* (min). After 40 min at adsorption equilibrium, the nanocomposite reached the maximum adsorption value of 94.86 mg/g. The adsorption of Ni(II) onto the LNC/MMT nanocomposite involves the following steps. First, the Ni(II) ions adsorb onto the surface of the LNC/MMT nanocomposite, where it reacts with active functional groups, thus accelerating the adsorption rate. Second, the Ni(II) ions diffuse into the interior through microporous surface channels on the LNC/MMT nanocomposite, decreasing the adsorption rate. Finally, adsorption equilibrium is reached.

The adsorption kinetic curve of the LNC/MMT nanocomposite was modeled by fitting the pseudo-first-order (3) and the pseudo-second-order adsorption kinetic equation (4) [29]:

$$\text{log}(q_{e}-q_{t})=\text{log}\;q_{e}-\frac{k_{1}t}{2.303}$$ (3)

$$\frac{t}{q_{t}}=\frac{1}{k_{2}{q_{e}}^{2}}+\frac{t}{q_{e}}$$ (4)

where *q_e_* (mg/g) is the amount of Ni(II) adsorbed upon reaching equilibrium, *q_t_* (mg/g) is the amount of Ni(II) adsorbed at time *t* (min), and *k_1_* (min^−1^) and *k_2_* [g·(mg/min)^−1^] are the rate constants of the pseudo-first-order and pseudo-second-order adsorption kinetic equations, respectively.

**Fig 6:**
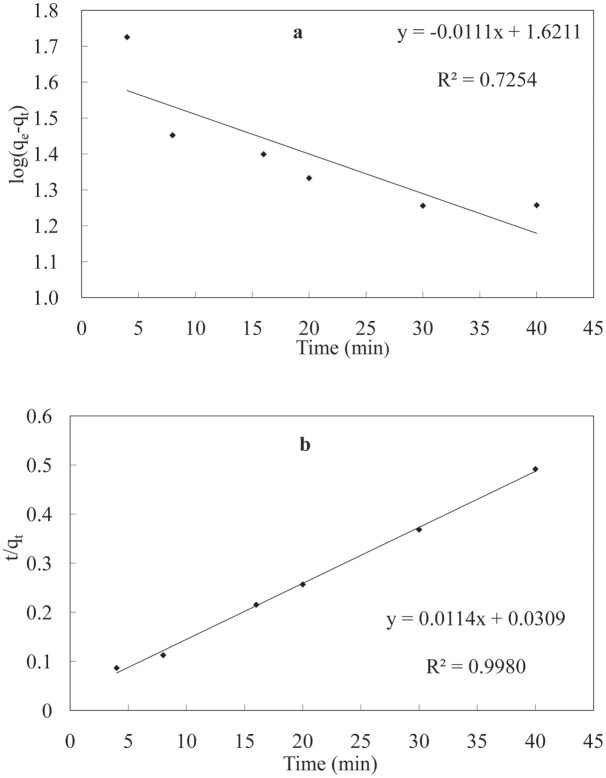
Pseudo-first-order (a) and pseudo-second-order (b) adsorption kinetic equations fitting curves of the experimental data. 70°C; initial Ni(II) concentration 0.0032 mol/L, pH = 6.8.

The results of the adsorption kinetics and fitting models are listed in Table 1 and Fig. 6. For the adsorption of Ni(II) ions by the LNC/MMT nanocomposite, the fitting correlation coefficient (*R* ^2^) of the pseudo-second-order adsorption kinetic equation was higher than that of the pseudo-first-order kinetic equation. The adsorption amount obtained experimentally at equilibrium was similar to that determined from the pseudo-second-order adsorption kinetic equation [30].

**Table 1:** Kinetic parameters for Ni(II) adsorption onto LNC/MMT.

**Models**	***q*_e_/mg/g**	***q*_ec_/mg/g**	***k***	**Correlation coefficient (*R*^2^)**
Pseudo-first-order	94.86	41.79	0.0256	0.7254
Pseudo-second-order	94.86	87.72	0.0042	0.9980

### Isotherm Studies

**Fig 7:**
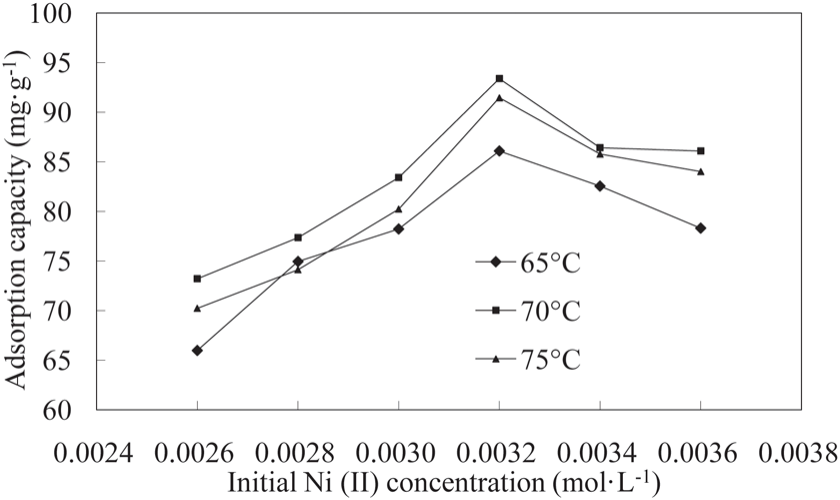
Effects of initial Ni(II) concentration on adsorption by LNC/MMT at various temperatures.

The study of equilibrium curves is very important for understanding the design of an adsorption system. In this study, two adsorption isotherms, the Langmuir and Freundlich adsorption isotherm models, were used to describe the obtained equilibrium data. Fig. 7 shows the adsorption capacity of the LNC/MMT nanocomposite at different initial Ni(II) concentrations at adsorption temperatures of 65, 70, and 75°C. Isothermal adsorption curves were plotted using the equilibrium adsorption amount *q_e-_* and the adsorption equilibrium concentration *C_e_*. As shown in Fig. 7, the adsorption equilibrium amount on the LNC/MMT nanocomposite surface was enhanced by increasing the initial Ni(II) concentration. Moreover, the degree of increase was higher at lower concentrations and decreased with increasing initial Ni(II) concentration, most likely because of the amount of active adsorption sites on the LNC/MMT nanocomposite. At a low initial Ni(II) concentration, the LNC/MMT nanocomposite would have sufficient active adsorption sites to interact with all Ni(II) ions; however, at initial Ni(II) concentrations beyond a critical saturation level, the active adsorption sites on the adsorbent surface were mostly occupied by Ni(II) ions, further limiting the adsorption.

The isothermal adsorption curves were plotted and fitted employing the Langmuir (5) and Freundlich (6) equations [31]:

$$\frac{C_{e}}{q_{e}}=\frac{1}{bq_{max}}+\frac{C_{e}}{q_{max}}$$ (5)

$$\text{ln}\;q_{e}=\text{ln}\;k_{f}+\frac{1}{n}\text{ln}\;C_{e}$$ (6)

where *b* (L/mg) is the Langmuir constant related to the adsorption capacity, *n* and *k_f_* are the Freundlich constants, *C_e_* (mol/L) is the concentration of Ni(II) at equilibrium, *q_max_* (mg/g) is the monolayer saturation adsorption, and *q_e_* (mg/g) is the adsorption capacity at equilibrium.

**Fig 8:**
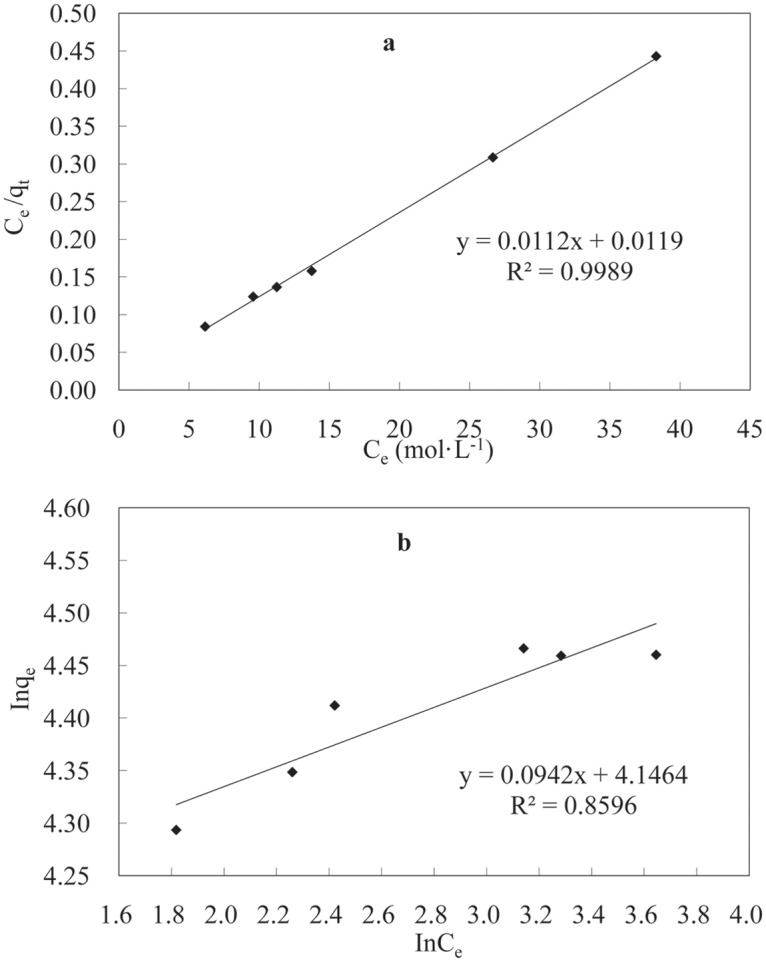
Langmuir (a) and Freundlich (b) isotherm equations fitting curves of the experimental data. T = 70°C, pH = 6.8, t = 40 min.

The fitting results from Fig. 8 are listed in Table 2. Comparisons of the *R* ^2^ values obtained for the fitting of the data collected at T = 70°C and pH = 6.8 reveal that the adsorption of Ni(II) ions by the LNC/MMT nanocomposite is consistent with Langmuir isothermal adsorption model; in addition, the *R* ^2^ value is 0.9989, indicating that the mechanism involves monolayer adsorption [32].

**Table 2:** Adsorption equilibrium constants obtained from Langmuir and Freundlich isotherms for Ni(II) adsorption onto LNC/MMT (T = 70°C, pH = 6.8, t = 40 min).

**Langmuir model**			**Freundlich model**		
***q*_max_ (mg/g)**	***b***	**Correlation coefficient (*R*^2^)**	***k_f_***	***1/n***	**Correlation coefficient (*R*^2^)**
94.86	0.9411	0.9989	63.206	0.0942	0.8596

### Characteristics of the Adsorption Mechanism

The surface structure of the LNC/MMT nanocomposite was directly related to the adsorption of Ni(II) ions. XRD and TEM were an effective method for the investigation of MMT and LNC/MMT structural characteristics. The surface microstructure of the LNC/MMT nanocomposite before and after the adsorption of Ni(II) ions was investigated by SEM and EDX. FTIR analysis was conducted to explore the active adsorption sites on the LNC/MMT nanocomposite surface to understand the fundamental reasons for the differences in the adsorption properties.

**Fig 9:**
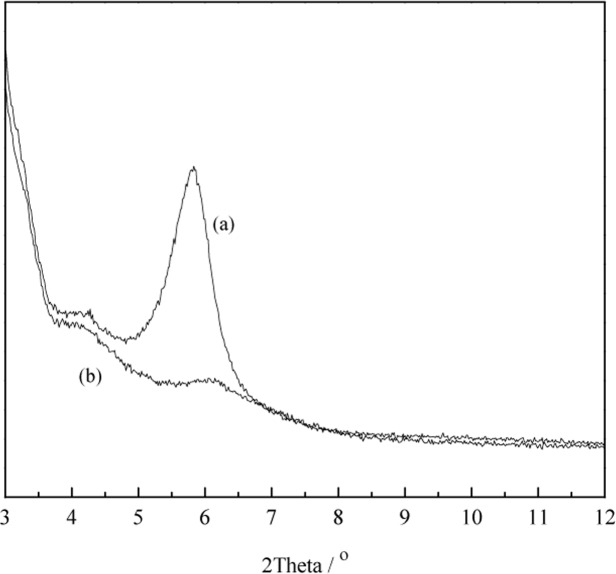
XRD patterns of MMT (a) and LNC/MMT nanocomposite (b).

**XRD analysis**. The powder XRD patterns of MMT and LNC/MMT were recorded to study the structure of the LNC-MMT nanocomposite. Fig. 9 illustrates the powder XRD patterns of MMT and the LNC-MMT nanocomposite. The powder XRD pattern of purified MMT (Fig. 9a) showed a typical diffraction peak at *2θ* = 5.83°. According to the Bragg equation [2*d*sin*θ* = *kλ* (*k* = 1, 2, 3, …)], this diffraction peak correlates to a basal spacing of *d* = 1.52 nm. After the MMT was intercalated with LNC (Fig. 9b), the typical diffraction peak of MMT shifted to a higher angle (*2θ* = 5.97°). Moreover, the intensity of the diffraction peak decreased and even disappeared which indicated the formation of a flocculated-intercalated nanostructure. Based on the XRD results, almost all of the LNC intercalated into the MMT interlayer, resulting in the destruction of the structure of MMT. XRD was an effective method for investigating the intercalation of LNC into MMT.

**Fig 10:**
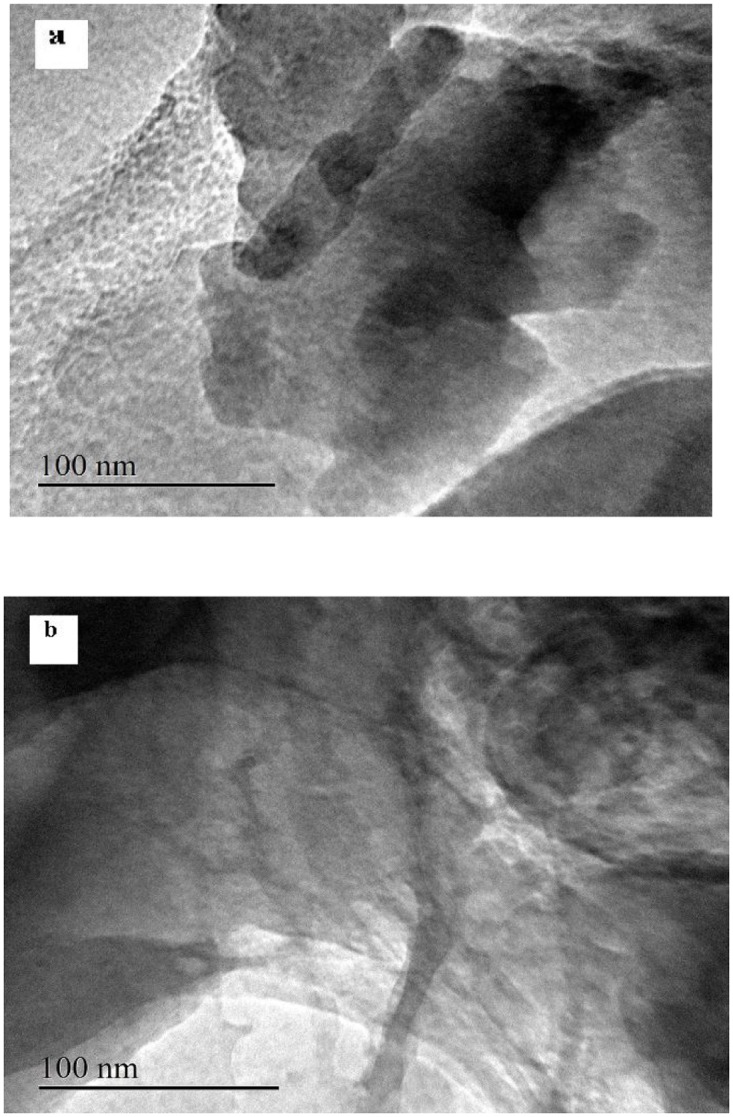
TEM images of purified MMT (a) and LNC/MMT (b).

**TEM analysis**. Fig. 10 shows the TEM images of purified MMT (a) and LNC/MMT (b). As shown in Fig. 10, compared with the stacks of multilayers of MMT (Fig. 10a), those of LNC/MMT (Fig. 10b) were thin and dispersive, which indicated that dispersion of MMT nanoplatelets was achieved via clay modification by LNC. Based on the XRD and TEM results, almost all of the LNC intercalated into MMT interlayers, with destruction of the crystalline structure of MMT, resulting in a different Ni(II) adsorption capacity for the LNC/MMT nanocomposite. These results will be discussed in detail in the following sections.

**Fig 11:**
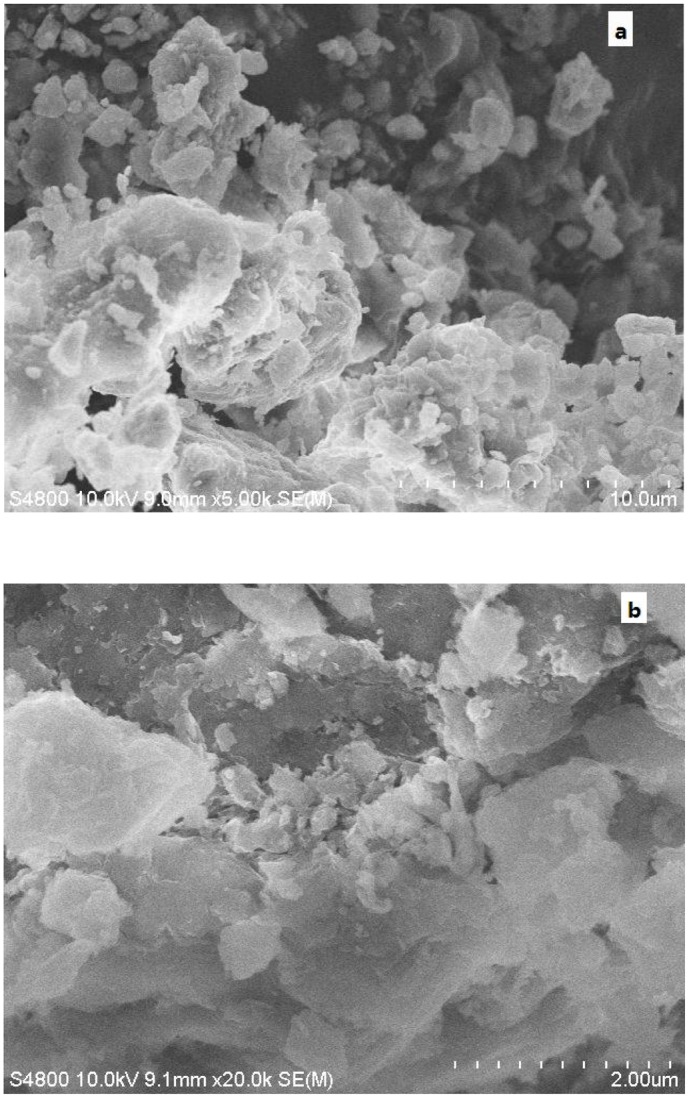
SEM image of LNC/MMT (a) and LNC/MMT after adsorption Ni(II) (b).

**SEM analysis**. The SEM images of LNC/MMT before and after Ni(II) adsorption are shown in Fig. 11. The SEM images of the LNC/MMT nanocomposite before (Fig. 11a) and after the adsorption of Ni(II) ions (Fig. 11b) showed curly or irregular clusters of polymerized sheet-stacked dispersions on the flat surface of the LNC/MMT nanocomposite; the presence of these clusters indicates that LNC reacted with MMT, destroying the nanolamellar structure of MMT that spread into the LNC matrix because of its microporous structure, thereby increasing the contact area for the adsorption of Ni(II) ions. After the adsorption of Ni(II) ions, the LNC/MMT nanocomposite surface was evenly packed with Ni(II) ions and the sheet-stacking structure was no longer apparent, indicating that the active sites for adsorbing Ni(II) ions existed mainly on the protruding tips of the sheet-stacked surface. Ni(II) ions functioned as a bridge among the connected surface active sites, resulting in a smooth surface, which further indicates the strong interactions of Ni(II) ions with the LNC/MMT nanocomposite surface. The adsorption was mainly dominated by chemical interactions [33].

**Fig 12:**
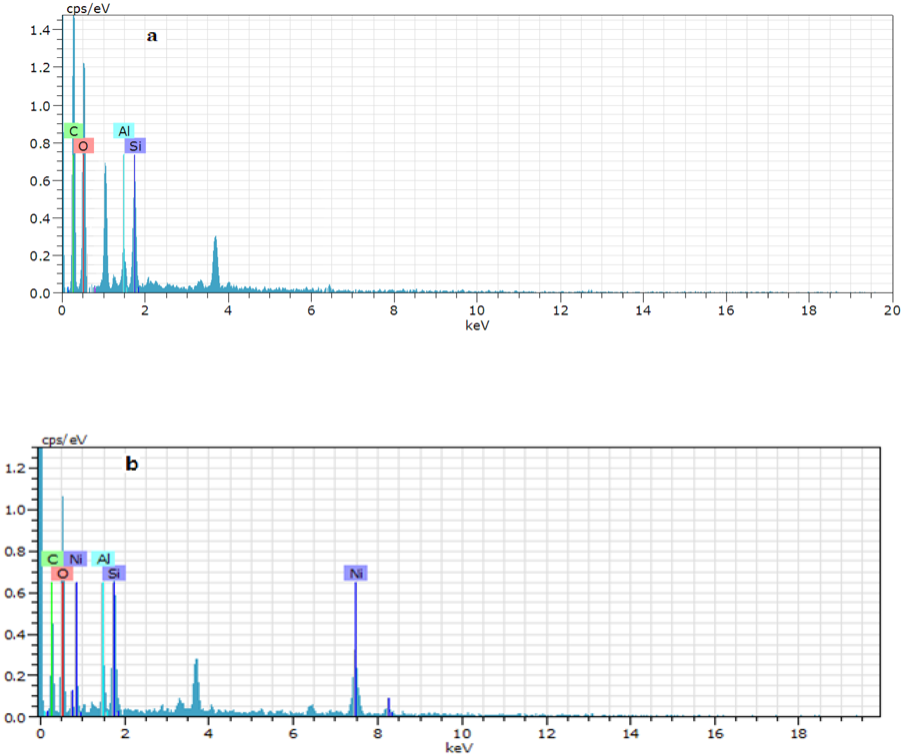
EDX results for fresh LNC/MMT (a) and after adsorption Ni(II) (b).

**EDX analysis**. The LNC/MMT was analyzed using EDX to explain the mechanisms of Ni(II) adsorption onto LNC/MMT. As shown in Fig. 12, the EDX results for fresh LNC/MMT nanocomposites (Fig. 12a) indicated the presence of C (46.69%), O (47.86%), Si (3.30%), and Al (1.37%). In the EDX spectrum of the LNC/MMT nanocomposite after Ni(II) adsorption (Fig. 12b), two new Ni(II) peaks emerged; the Ni content was determined to be 19.37%. This result confirmed the presence of Ni(II) ions on the LNC/MMT nanocomposite surface. The protruding tips were not evenly distributed, indicating that only selected functional groups are involved in the adsorption of Ni(II) ions.

**Fig 13:**
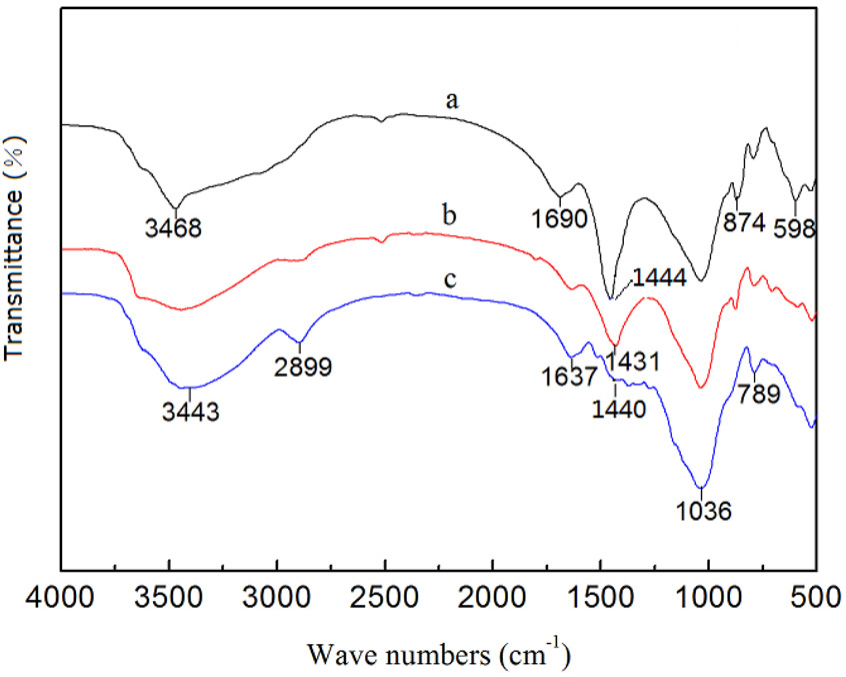
FTIR spectra of LNC/MMT(a), LNC/MMT after the adsorption (b), after the desorption (c).

**FTIR analysis**. To determine the primary functional groups of the LNC/MMT nanocomposite involved in the adsorption of Ni(II) ions, FTIR spectra were recorded for the LNC/MMT nanocomposite after the adsorption and desorption of Ni(II) ions (Fig. 13). The results show that the chemical structure of the LNC/MMT underwent a significant change after Ni(II) adsorption. As shown in Fig. 13, the adsorption bands at 3468 cm^−1^ (Fig. 13a) were attributed to the intramolecular O—H stretching vibration absorption peak as well as to the characteristic absorption band of intermolecular hydrogen bonding between phenol and alcohol molecules. This peak shifted to a lower wavenumber, 3443 cm^−1^ (Fig. 13b), after the adsorption of Ni(II) and exhibited a band in the spectrum, indicating that some of the O—H and corresponding hydrogen bonds were broken to react with Ni(II); this band weakened after desorption. The characteristic adsorption band at 1690 cm^−1^ (Fig. 13a), which corresponds to the asymmetric stretching vibration of the C = O bond in carboxylic acids, weakened significantly after the adsorption of Ni(II) and appeared again at a lower wavenumber of 1637 cm^−1^ after the desorption of Ni(II) (Fig. 13c). The vibration absorption peak of the carboxyl O—H bond, which was originally located at 1444 cm^−1^ (Fig. 13a), weakened after Ni(II) adsorption and shifted to 1431 cm^−1^ (Fig. 13b); this peak reappeared at 1440 cm^−1^ (Fig. 13c) after Ni(II) desorption. Moreover, the absorption band at 874 cm^−1^ (Fig. 13a), which represents the stretching vibration absorption of the aromatic and phenol C—H stretching bond, moved to a lower wavenumber after Ni(II) absorption and then shifted back to 789 cm^−1^ (Fig. 13c) after Ni(II) desorption. On the basis of these analyses, we concluded that the acidic protons of the hydroxyl and carboxyl groups of the LNC/MMT structure were substituted with Ni(II) after adsorption. Slight changes in intensities were observed in the corresponding vibration absorption bands of the reactive functional groups. It can be suggested that the adsorption of Ni(II) mainly relies on chemical adsorption. Ni(II) formed complexes with organic ligands because of its unique structure with empty orbitals in the outer valence shell. Ni(II) chelated with –COOH, –OH on the LNC/MMT nanocomposite. This results confirmed that binding sites existed on the surface of the LNC/MMT nanocomposite that could couple with Ni(II). Moreover, the adsorption bands shifted upon Ni(II) adsorption. Furthermore, after desorption of Ni(II), the FTIR spectrum of the absorbent nearly coincided with that of the original LNC/MMT nanocomposite. Therefore, the basic structure and properties of the LNC/MMT remained relatively stable during the process of Ni(II) adsorption and desorption, which suggests that it may be applicable as an effective renewable adsorbent.

### Desorption and Regeneration Studies

The regeneration of the LNC/MMT nanocomposite after adsorption is critical for its economic viability in practical applications [34, 35]. The desorption properties of the LNC/MMT nanocomposite saturated with adsorbed Ni(II) were investigated. The effects of the desorption reagent, HNO_3_ concentration, desorption temperature, and desorption time on the desorption capacity were investigated.

**Fig 14:**
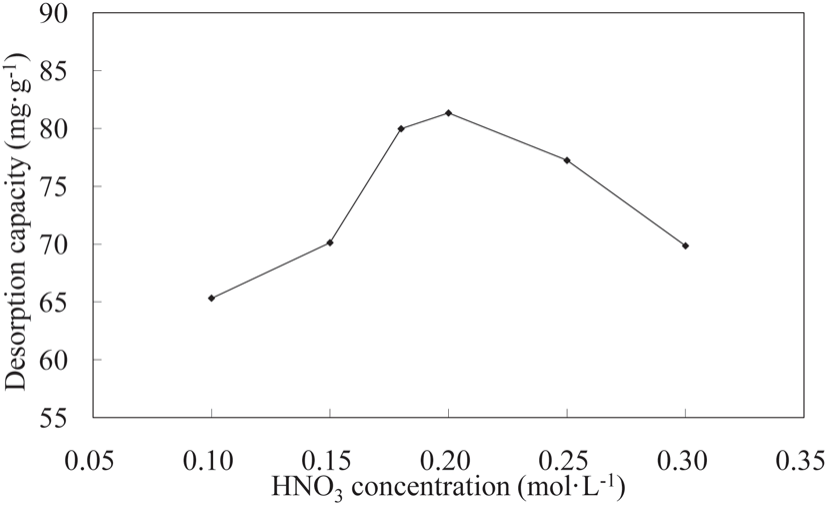
Effect of HNO_3_ concentration on desorption capacity of LNC/MMT. Desorption experiments-sample dose, 0.1000 g; temperature, 60°C; time, 30 min.

**Effect of HNO3 concentration on desorption**. The effects of different HNO_3_ concentrations on Ni(II)-loaded LNC/MMT vs. desorption capacity are shown in Fig. 14. The results indicate that the desorption capacity of the LNC/MMT nanocomposite first increased and then decreased with increasing HNO_3_ concentration. This observation is likely attributable to the increase in acid concentration leading to the accumulation of H^+^ in solution, thus increasing the concentration gradient of Ni(II) and H^+^ and resulting in an increased driving force for ion-exchange, favoring the desorption process. However, a higher concentration of HNO_3_ would dramatically increase the H^+^ concentration in solution and thus may enhance electrostatic repulsion among Ni(II) ions, inhibiting Ni(II) desorption [26]. The maximum desorption capacity of the LNC/MMT nanocomposite reached 81.34 mg/g at an HNO_3_ concentration of 0.2 mol/L.

**Fig 15:**
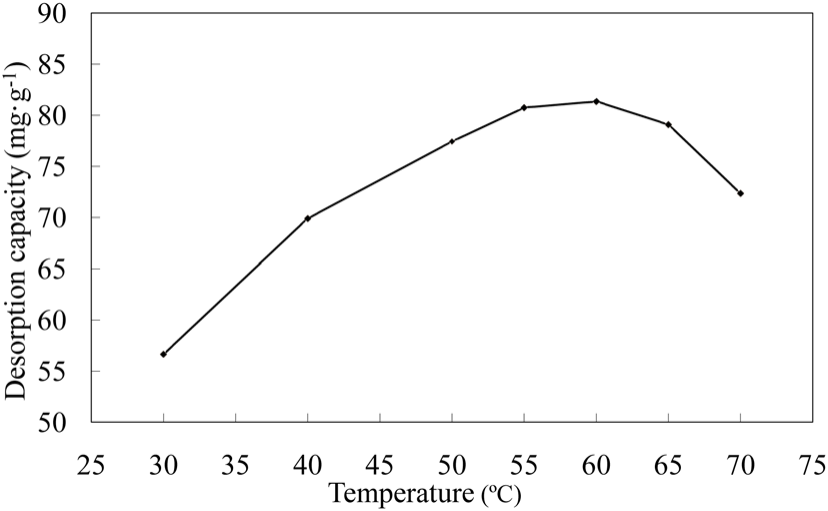
Effect of temperature on desorption capacity of LNC/MMT. Desorption experiments-sample dose, 0.1000 g; HNO_3_ concentration, 0.2 mol/L; time, 30 min.

**Effect of temperature on desorption**. The effects of different desorption temperatures on the Ni(II)-loaded LNC/MMT nanocomposite desorption capacity are shown in Fig. 15. The results indicate that the desorption capacity of the LNC/MMT nanocomposite first increased and then decreased with increasing temperature. The decrease in desorption capacity was attributed to the higher temperature, which may increase the desorption capacity [36]. Therefore, 60°C was selected for subsequent experiments.

**Fig 16:**
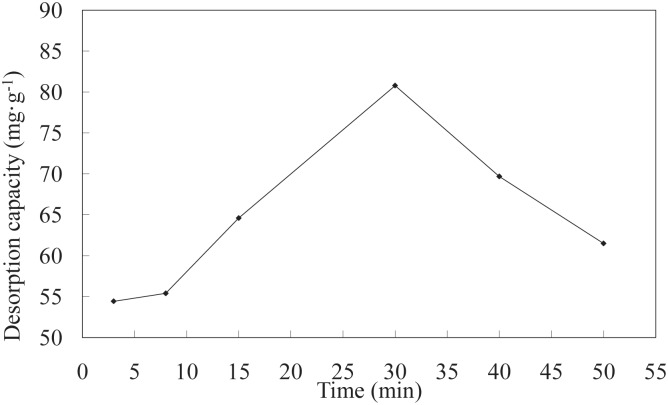
Effect of time on desorption capacity of LNC/MMT. Desorption experiments-sample dose, 0.1000 g; HNO_3_ concentration, 0.2 mol/L; temperature, 60°C.

**Effect of time on desorption**. The effects of different desorption times on the Ni(II)-loaded LNC/MMT nanocomposite are shown in Fig. 16. The results indicate that the desorption capacity of the LNC/MMT nanocomposite first increased and then decreased with increasing desorption time. This phenomenon was consistent with the occurrence of holes [37] produced under ultrasonic conditions. After a certain ultrasonic oscillation time, the concentration of holes in solution reached saturation, and the LNC/MMT nanocomposite reached a maximum desorption level after an ultrasonic desorption time of 30 min. The LNC/MMT nanocomposite can potentially be regenerated and recycled.

**Recycling and reusability of LNC/MMT**. The reuse of the LNC-MMT nanocomposite would decrease processing costs and may open the possibility of recovering the LNC/MMT nanocomposite after Ni(II) extraction from wastewater. Thus, the ability to recycle the LNC-MMT nanocomposite is a very important consideration with respect to its practical applications. The reusability of the LNC-MMT nanocomposite was investigated by six consecutive adsorption-desorption processes. The effects of the number of cycles on the Ni(II) ions adsorption/desorption capacity are shown in Table 3. These results indicate that the LNC/MMT nanocomposite could be recycled up to five times while retaining optimal adsorption-desorption conditions. The adsorption and desorption capacities for the 6^th^ process were 50.16 mg/g and 38.90 mg/g, respectively. The results of this study indicate that the LNC/MMT nanocomposite could be used as an adsorbent in practical applications for the removal of Ni(II). Therefore, the LNC-MMT nanocomposite has potential applications as a high-performance, inexpensive, and recyclable green adsorbent for wastewater treatment.

**Table 3:** LNC/MMT adsorption/desorption capacities for Ni(II) after six consecutive cycles.

**Recycle times**	**1st**	**2nd**	**3rd**	**4th**	**5th**	**6th**
Adsorption *Q*_e_ (mg/g)	94.86	92.67	90.18	88.10	85.34	50.16
Desorption *Q*_e_ (mg/g)	81.34	76.88	73.52	70.43	65.27	38.90

## Conclusions

A new LNC/MMT nanocomposite was prepared by chemical intercalation. The results of this study showed that the synthesized LNC/MMT nanocomposite could be used effectively for the adsorption of Ni(II) ions from aqueous solutions. The maximum Ni(II) ion adsorption capacity for the LNC/MMT nanocomposite reached 94.86 mg/g under an initial Ni(II) concentration, pH, adsorption temperature, and adsorption time of 0.0032 mol/L, 6.8, 70°C, and 40 min, respectively. The adsorption kinetics and isotherms were well fit to both the pseudo-second-order adsorption kinetic equation (*R* ^2^ = 0.9980) and the Langmuir isothermal adsorption models (*R* ^2^ = 0.9989), respectively, indicating that within the experimental range, the adsorption equilibrium was mainly dominated by monolayer chemical adsorption.

The effects on the desorption capacity of the Ni(II)-loaded LNC/MMT nanocomposite were investigated using HNO_3_ as the desorption reagent in ultrasonic oscillation treatments. The optimum desorption conditions are based upon a concentration of HNO_3_, a desorption temperature, and a desorption time of 0.2 mol/L, 60°C, and 30 min, respectively. Under the optimum conditions, the maximum desorption capacity was 81.34 mg/g.

The adsorption-desorption experiments showed that the adsorption and desorption capacities of the LNC/MMT nanocomposite remained at a relatively high level after five adsorption-desorption cycles. Therefore, the LNC/MMT nanocomposite could be recycled if used to remove Ni(II) ions from Ni(II)-polluted wastewater.
